# cRGD-Functionalized Silk Fibroin Nanoparticles: A Strategy for Cancer Treatment with a Potent Unselective Naphthalene Diimide Derivative

**DOI:** 10.3390/cancers15061725

**Published:** 2023-03-11

**Authors:** Valentina Pirota, Giovanni Bisbano, Massimo Serra, Maria Luisa Torre, Filippo Doria, Elia Bari, Mayra Paolillo

**Affiliations:** 1 Department of Chemistry, University of Pavia, Viale Taramelli 10, 27100 Pavia, Italy; 2 Department of Drug Sciences, University of Pavia, Viale Taramelli 12, 27100 Pavia, Italy; 3 Department of Pharmaceutical Sciences, University of Piemonte Orientale, Largo Donegani 2/3, 28100 Novara, Italy; 4 PharmaExceed S.r.l., Piazza Castello, 19, 27100 Pavia, Italy

**Keywords:** RGD, silk fibroin nanoparticles, integrin antagonist, naphthalene diimides, anticancer, active targeting, brain tumours, NDI, drug delivery, cyclic pentapeptides

## Abstract

**Simple Summary:**

For many types of cancer, chemotherapy is still widely used, but it can cause serious side effects because it kills all rapidly growing cells, not just the cancerous ones. In this paper, a nanoparticle-based drug delivery system has been designed to selectively deliver a highly cytotoxic drug to tumor cells. The nanoparticles, obtained from a protein called silk fibroin, have been loaded with a naphthalene diimide derivative, namely NDI-1, which kills cells by affecting their DNA. The surface of the silk fibroin nanoparticles has been decorated with cyclopentapeptides incorporating the Arg-Gly-Asp sequence (cRGDs) to specifically target the tumor cells, thus reducing the harm to healthy cells and minimizing side effects.

**Abstract:**

Developing drug delivery systems to target cytotoxic drugs directly into tumor cells is still a compelling need with regard to reducing side effects and improving the efficacy of cancer chemotherapy. In this work, silk fibroin nanoparticles (SFNs) have been designed to load a previously described cytotoxic compound (NDI-1) that disrupts the cell cycle by specifically interacting with non-canonical secondary structures of DNA. SFNs were then functionalized on their surface with cyclic pentapeptides incorporating the Arg-Gly-Asp sequence (*c*RGDs) to provide active targeting toward glioma cell lines that abundantly express ανβ3 and ανβ5 integrin receptors. Cytotoxicity and selective targeting were assessed by in vitro tests on human glioma cell lines U373 (highly-expressing integrin subunits) and D384 cell lines (low-expressing integrin subunits in comparison to U373). SFNs were of nanometric size (d_50_ less than 100 nm), round shaped with a smooth surface, and with a negative surface charge; overall, these characteristics made them very likely to be taken up by cells. The active NDI-1 was loaded into SFNs with high encapsulation efficiency and was not released before the internalization and degradation by cells. Functionalization with *c*RGDs provided selectivity in cell uptake and thus cytotoxicity, with a significantly higher cytotoxic effect of NDI-1 delivered by cRGD-SFNs on U373 cells than on D384 cells. This manuscript provides an in vitro proof-of-concept of *c*RGD-silk fibroin nanoparticles’ active site-specific targeting of tumors, paving the way for further in vivo efficacy tests.

## 1. Introduction

Finding novel and effective cancer treatments is a key issue on a global scale [1]. Despite an increase in the number of cancer treatment options and the significant increase in the therapeutic efficacy for various malignant tumors, much remains to be done. In this regard, chemotherapy is still widely exploited as a cancer treatment method; it makes use of cytotoxic drugs to disrupt the cell cycle and cause apoptosis by directly targeting the key proteins required for cell division or interfering with DNA [2]. As in many cancer cells the telomere length is maintained due to the overexpression of the reverse transcriptase enzyme telomerase, a specifical action mechanism for some cytotoxic drugs consists of sequestering the single-stranded telomeric DNA, i.e. the enzyme’s substrate, by inducing and/or stabilizing the folding of G-quadruplex structures [3,4]. In this way, without its substrate, the enzyme cannot work, and the telomere length can again progressively shorten, ultimately leading to senescence and apoptosis, as with non-malignant cells. Many monomeric and dimeric naphthalene diimide derivatives (NDIs) have been shown to behave according to this mechanism, strongly inducing/stabilizing G-quadruplexes at the telomeric level, thus acting as potent anticancer agents [5,6,7,8,9].

Although some NDIs have also reached clinical trials in humans [10], they can cause severe side effects due to indiscriminate cell entry, including non-malignant ones, with a very low therapeutic window [11,12]. Many synthetic efforts have been made to modify the naphthalene core to increase the selectivity towards malignant cells, in some cases achieving promising results [12]. However, according to most of the cancer-related research in the past few decades, it is possible to overcome this issue by developing drug delivery systems that selectively target these molecules to tumors. In this regard, many nanoparticle (NP)-based drug delivery systems have been designed to precisely target tumor cells after intravenous administration through their carrier effect and the positioning effect of a targeting substance. At first, depending on their size and characteristics, e.g., shape and surface charge, NPs leave the systemic blood circulation and reach and accumulate in the tumor by passive targeting due to the well-known enhanced permeation and retention effect (EPR) [13]. The selective uptake of NPs can then be achieved by functionalizing the surface of the nanoparticles with molecules that bind to ligands uniquely expressed on the target cells.

The integrin superfamily members such as α4β1, α5β1, α6β4, ανβ3, and ανβ5 [14] are ideal for this type of strategy because of their overexpression on the surface of many tumor cells and neoangiogenic vessels. Integrins are among the most representative and studied receptors involved in cellular adhesion processes and, through binding with their endogenous ligands, they mediate cell-cell and cell-ECM (extracellular matrix) interactions. In particular, αvβ3 and αvβ5 integrin subtypes are considered interesting targets because of their important role in many biological processes and diseases, such as embryonic development [15,16] and osteoporosis [17]; furthermore, they have been found to be overexpressed in many cancer types, such as high-grade gliomas [18,19,20]. Integrin receptors, which can undergo internalization following the interaction with their ligands [21,22], are involved in metastasis-related ECM modifications through the modulation of matrix metalloproteinases (MMP) activity [23,24,25,26]. Moreover, activated integrins were also found in tumor-secreted exosomes, which contribute to cancer’s horizontal progression [27,28], and are implicated in preparing the premetastatic niche in distant tissues.

Peptidic and peptidomimetic compounds incorporating the Arg-Gly-Asp (RGD) sequence are known integrin antagonists with potential applications in anticancer therapy [29,30]. If linked to a nanoparticle, RGD derivatives can then be exploited to selectively deliver the nanosystem into the tumor cells [31,32,33]. As a result, once internalized, the drug is released following NP degradation, thereby selectively killing malignant cells only. Many of the advantages provided by NPs in cancer treatment, such as the reduction of side effects and drug resistance, improved drug efficacy and improved pharmacokinetics, are achieved by enhancing permeability and retention into tumor tissues through these passive and active accumulation mechanisms, together with improved bioavailability (by increasing the solubility of poorly soluble drugs and by allowing their delivery into circulation) [34].

Although this assumption works very well theoretically, putting it into practice is a whole different thing. To develop a drug delivery system characterized by all these properties, choosing a biocompatible and biodegradable material with good mechanical properties and endowed with functional groups that allow easy functionalization is mandatory. Among the most employed materials for the preparation of NPs, silk fibroin, a protein extracted from *Bombyx mori* cocoons, is one of the most promising, as it possesses all the ideal characteristics mentioned above. The literature is rich in examples where silk-fibroin nanoparticles (SFNs) have been used to deliver different biologically active compounds, including cytotoxic ones, and they have also been functionalized to provide active targeting [35,36,37,38,39]. In our previous work, we reported the derivatization of SFNs with RGD-based cyclic pentapeptides (*c*RGDs) [40] capable of selectively binding αvβ3 and αvβ5 integrin subtypes, thus being used to achieve a selective accumulation of NPs in malignant cells. Moreover, opportunities to transfer SFNs from bench to bedside, even though they still need to be addressed, are encouraging due to the possibility of producing them with good manufacturing practice (GMP) and large-scale manufacturing processes. This adds to an already delineated horizon with regard to quality control and batch release requirements, biocompatibility, biodegradability, and safety tests that must be performed [41].

Given these premises, this work aimed, for the first time, to exploit SFNs to encapsulate and deliver NDI-1, a highly cytotoxic tetrasubstituted naphthalene diimide derivative. The selective active targeting was provided by functionalizing the surface of SFNs with RGD-based cyclopentapeptides. Both loaded and unloaded, naked, or functionalized SFNs were fully characterized by size and size distribution, zeta potential, physical-chemical properties, morphology, and ultrastructure. Finally, formulations were tested to demonstrate the selective cytotoxic effect of NDI-1 loaded into SFNs functionalized with *c*RGDs towards tumor cell lines that highly express αvβ3 and αvβ5 integrin receptors.

## 2. Materials and Methods

### 2.1. Materials and Equipements

Solvents and reactants for the synthesis of the active compound NDI-1 were purchased from Carlo Erba, Zentek, and Merck (Milan, Italy), and were used as supplied without further purification. Yields were calculated for compounds purified by HPLC chromatography and judged homogeneous by thin-layer chromatography, NMR, and mass spectrometry. Microwave irradiation was performed with a Discover microwave synthesizer (CEM, Cologno al Serio, Italy). Reactions were monitored by analytic HPLC using an Agilent system SERIES 1260 (Agilent Technologies, Santa Clara, CA, USA) with XSelect^®^ HSS C18 column (2.5 µm, 4.6 × 50 mm). The following method was used: isocratic gradient over 2 min 95% of H_2_O + 0.1% trifluoroacetic acid (5% acetonitrile), gradually to 40% aqueous solvent over 6 min, then isocratic flow for 4 min (λ = 256 nm; flow rate 1.4 mL/min). Purification of the final product was carried out by using an Agilent Technologies 1260 Infinity preparative HPLC (Agilent Technologies, Santa Clara, CA, USA) provided with a diode array UV-vis detector, using a Waters XSelect^®^ CSH Prep C18 OBDTM column (5 µm, 100 × 30 mm) at 30 mL/min flow rate. Aqueous solvent: 0.1% trifluoroacetic acid in water; organic solvent: acetonitrile; gradient: 95% aqueous for 2 min after injection, gradually to 40% aqueous over 16 min (λ = 254 nm, 600 nm). UPLC-MS data were recorded employing a UPLC system (Thermo Finnigan, San Jose, CA, USA) supplied with a BEH Acquity UPLC column (1.7 μm) 2.1 × 50 mm, and an LCQ ADV MAX ion-trap mass spectrometer, with an ESI ion source. In addition, ^1^H- and ^13^C-NMR spectra were recorded on a Bruker Avance 300 MHz (BRUKER, Billerica, MA, USA).

*Bombyx mori* cocoons were kindly donated by Nembri Industrie Tessili S.r.l., Capriolo, Italy. All the reagents for preparing and characterizing SFNs were purchased from Merck (Milan, Italy) and used without further purification. All the dialyses were performed using cellulose tubes purchased from Spectrum Laboratories (Milan, Italy).

Human glioma cell lines (U373 and D384) were purchased from the European Collection of Authenticated Cell Cultures Cell Bank (ECACC, Salisbury, UK). All reagents used for cell cultures were purchased from Euroclone (Milan, Italy).

### 2.2. Synthesis of NDI-1

**Scheme 1:**
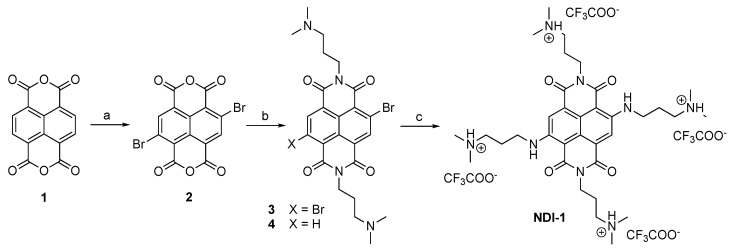
Synthesis of NDI-1: (a) dibromoisocyanuric acid, H_2_SO_4_, reflux, overnight (yield 85%); (b) 2.5 equivalents of N,N-dimethyl-1,3-propanediamine, glacial CH_3_COOH, reflux, 30 min; (c) neat N,N-dimethyl-1,3-propanediamine, microwaves, 120 °C, 15 min, 250 psi, 200 W (step b and c overall yield 44%).

The synthesis and characterization of NDI-1 were already reported in the literature [42]; nevertheless, here it was synthesized following a more efficient protocol (Scheme 1). Commercial naphthalene dianhydride 1 (2.0 g, 7.44 mmol) and dibromoisocyanuric acid (3.2 g, 11.12 mmol) were dissolved in 50 mL of sulphuric acid (98%) and refluxed overnight under stirring (Scheme 1, step a). The mixture was poured onto ice, inducing the precipitation of a yellow solid. It was filtered, washed with water (2 × 25 mL), resuspended in dichloromethane, and dried under vacuum obtaining 2 (Scheme 1, step a) as a yellow powder (yield 85%). Compound 2 (0.5 g, 1.17 mmol) was resuspended in glacial acetic acid (70 mL), and the solution was brought to reflux. N,N-dimethyl-1,3-propanediamine (0.367 mL, 2.93 mmol) was added dropwise, and the solution was maintained to reflux for 30 min (Scheme 1, step b). The mixture was cooled to room temperature and poured in small portions into sodium carbonate saturated ice by shaking the mixture vigorously. Further sodium carbonate was added until pH = 7–8 was reached. The aqueous solution was then extracted in dichloromethane (3 × 50 mL), and the organic solvent was dried over sodium sulfate, filtered, and evaporated under reduced pressure. The mono- and dibromo adducts 3 and 4 were obtained as a solid orange mixture and used without further purification. The rate between di- and monobromo adducts was evaluated by analytic HPLC recording the chromatogram at 256 nm (70% of 3 and 30% of 4). The mixture of 3 and 4 (250 mg) was suspended in neat N,N-dimethyl-1,3-propanediamine (2 mL) in a closed vessel and stirred at 120 °C for 15 min (250 psi, 200 W) under microwave assistance to perform the final aromatic nucleophilic substitution reaction (Scheme 1, step c). The amine solvent was then evaporated under a vacuum, and the crude mixture composed by the desired product and the monoamine substituted NDI arising from 4 (not reported in Scheme 1) was purified by preparative HPLC. The desired NDI-1 was obtained as a blue tetracationic TFA salt in 44% yield over two steps (b and c). NMR spectra are consistent with those reported in the literature [42], and analytic HPLC confirmed the purity of NDI-1 equal to 96.2% (r.T. = 4.996 min) (see Figure S1 in the Supplementary Materials). 

### 2.3. Preparation and Functionalization of Silk-Fibroin Nanoparticles (SFNs)

#### 2.3.1. Silk-Fibroin Nanoparticles Preparation

Silk fibroin nanoparticles were prepared according to the procedure previously reported by our group [40]. Briefly, *Bombyx mori* cocoons were cut into 1 × 1 cm sections and boiled in 100 °C water with 0.02 M Na_2_CO_3_ for 30 min to solubilize the sericin. The fibroin fibers were then washed with deionized water, left to dry at room temperature, and then solubilized in LiBr 9.3 M at 60 °C for 4 h. The fibroin solution was filtered through a 70 μm nylon mesh, placed into dialysis cellulose tubes (3–5 kDa Molecular Weight Cut-Off, MWCO), dialyzed against distilled water for 72 h at room temperature, and diluted to reach a 1.5% *w/v* concentration. NDI-1 was solubilized in the fibroin solution at the final concentration of 0.31 mg/mL, and the mixture was then desolvated in acetone (fibroin:acetone ratio 1:5). Unloaded SFNs were prepared following the same procedures but without adding NDI-1. SFNs and SFNs-NDI-1 were placed into dialysis cellulose tubes (3–5 kDa Molecular Weight Cut-Off, MWCO) and dialyzed against distilled water for 72 h at room temperature. The nanoparticles were then freeze-dried at −46 °C with 0.28 mbar pressure (LIO-5PDGT, Cinquepascal, Milan, Italy).

#### 2.3.2. Derivatization Protocol

The synthesis of the *c*RGD derivative carrying an azide moiety (N3-*c*RGD) was performed by following the protocol reported in our previous works [40,43,44].

The surface of both unloaded (SFNs) and loaded (SFNs-NDI-1) nanoparticles was modified using the same functionalization strategy. SFNs or SFNs-NDI-1 (80.0 mg, Lys: 80 nmol/mg) were suspended in a phosphate buffer (8 mL, pH = 7.4) for 1 h to ensure their complete hydration, then a 10 mg/mL DMF solution of the alkyne linker was added (200 μL, 0.0096 mmol). After 24 h, the alkyne-functionalized nanoparticles were purified through dialysis against water at room temperature for 48 h. The alkyne-functionalized nanoparticles (60.8 mg, Lys: 80 nmol/mg) were then suspended in a phosphate buffer (3.1 mL, pH 7.4) for 1 h to ensure their complete hydration. A solution of N3-*c*RGD (4.7 mg, 0.0049 mmol) in DMSO (150 μL), a 0.01 M aqueous solution of CuSO_4_ (146 μL, 0.00146 mmol), and a 0.01 M solution of sodium l-ascorbate (730 μL, 0.00730 mmol) were then added to the reaction flask. After 72 h, the *c*RGD-functionalized nanoparticles were submitted to dialysis against distilled water at room temperature for 48 h.

### 2.4. Silk-Fibroin Nanoparticles Characterization

#### 2.4.1. Production Yield, Drug Loading, and Encapsulation Efficiency Evaluation

The yield % was calculated according to Equation (1):

Yield % = (total weight nanoparticles)/(weight of fibroin + weight of drug) × 100(1)

To quantify the NDI-1 loading into SFNs-NDI-1 or *c*RGD-SFNs-NDI-1, the nanoparticles were solubilized in LiBr 9.3 M at 60 °C for 4 h (2 mg/mL). The UV-visible spectra of the solutions were registered with an Agilent Cary 60 spectrophotometer (Agilent Technologies, Santa Clara, CA, USA), recording the absorbance from 200 to 900 nm wavelength interval at a scan rate of 50 nm/min and a slit width of 0.5 nm. Emission spectra were run on a Cary Eclipse Fluorescent Spectrophotometer (Agilent Technologies, Santa Clara, CA, USA) and recorded from 636 nm to 850 nm by exciting at 616 nm. A scan rate of 600 nm/min, averaging time 0.1 s, data interval 1 nm, excitation and emission slits 5 and 10 nm, were used to obtain the spectra as an average of 3 scans. A quartz cuvette of 10 mm path length was used. The characteristic absorbance and emission spectra of NDI-1 were used to correctly quantify its load in the silk fibroin nanoparticles. To calibration lines, NDI-1 (1.41 mg, 1.29 μmol) was suspended in a 100% *w/v* LiBr solution (1 mL) and heated at 60 °C for 4 h. Absorbance calibration lines were obtained by plotting the maxima of NDI-1 characteristic bands (365 nm and 616 nm) versus NDI-1 concentration from 0.25 μM to 2.5 μM. Using the same NDI-1 concentrations, the emission calibration lines were obtained by plotting the maximum emission bands at 657 nm, recorded with the slits opened at 10 and 5 nm. All measurements were conducted in triplicate by obtaining four calibration lines with R-square coefficients of determination equal to 0.998 ± 0.001, 0.997 ± 0.005, 0.999 ± 0.009, 0.99 ± 0.01, respectively. Finally, the drug loading was calculated according to Equation (2):

Drug loading % *w*/*w* = (total drug content)/(total weight nanoparticles) × 100 (2)

#### 2.4.2. Size Distribution and Zeta Potential

The mean diameter and the particle size distribution were measured for all the formulations by a NanoSight NS300 (Malvern Panalytical, Grovewood Rd, WR14 1XZ, Great Malvern, Worcestershire, UK). The nanoparticles were dispersed in water at 0.05 mg/mL and sonicated before the analysis. Five measurements of 90 s each were repeated for each sample, and the data were elaborated by the NTA software 3.0 provided by the manufacturer.

The zeta potential was measured using the Zetasizer nano ZS90 after the dispersion of SFNs at 1 mg/mL in KCl 1 mM. Each analysis was performed in triplicate.

#### 2.4.3. FT-IR Characterization

The infrared spectra of all nanoparticles were acquired with an Alpha II FT-IR spectrometer with a platinum Attenuated Total internal Reflectance (ATR) module (Bruker, Rosenheim, Germany), and data were elaborated with Opus 7.8 software.

#### 2.4.4. Morphological Evaluation by Scanning Electron Microscopy (SEM), Transmission Electron Microscopy (TEM), and Cryoelectron Microscopy (Cryo-EM)

For SEM analysis, the freeze-dried samples were placed on the stub and gold-sputter-coated under argon. The images were acquired by MIRA3 (Tescan, Brno, Czech Republic). TEM images were acquired by a JEOL JEM 1200 EX instrument. The samples were dispersed in water at the final concentration of 0.1 mg/mL; a drop of the sample suspension was then placed onto a 300-mesh nickel grid coated with carbon and let dry for 24 h at room temperature (25 °C) before analysis. Cryo-EM images were acquired by a Cryo transmission electron microscope Glacios (Thermo Fisher Scientific, Milan, Italy). The sample suspension (0.1 mg/mL) was placed on a grid (Quantifoil R 1.2/1.3 Cu 300), and the images were acquired.

### 2.5. In Vitro Biological Assays

#### 2.5.1. Cell Culture

U373 and D384 human glioblastoma cell lines were selected for the assays and purchased from the ECACC collection (Merk Life Science, Milan, Italy). The cells were cultured at 37 °C in DMEM supplemented with 10% *v/v* fetal bovine serum, 1 mM glutamax, 1% *v/v* antibiotics (penicillin-streptomycin), and 1 mM pyruvate (Merk Life Science, Milan, Italy) at 37 °C under a humidified atmosphere containing 5% CO_2_.

#### 2.5.2. Real-Time PCR

To assess integrin subunits expression in U373 and D384 cells, the mRNA expression of αv, α5, β1, β3, and β5 were evaluated as previously described [45]. In brief, RNA was extracted from the cell lines followed by a DNAse digestion step. The primers used for integrin subunits in quantitative real-time RT-PCR reactions were reported in a previous study [45] and are shown in Table 1. β-actin was used as a housekeeping gene. Experiments were performed on three different cell preparations, and each run was analyzed in duplicate. Data are expressed as ΔCq (difference between reference and housekeeping gene Cq) [45].

#### 2.5.3. Cytotoxic Activity Evaluation

Cell viability assays were performed using the MTS assay after 24 and 72 h treatments. The cells were seeded in a 96-well plate (5000 cells/well) and cultured with their culture media, as described in Section 2.5.1. At 24 h after plating, the medium was replaced by 100 μL of serum-free medium, and 24 h after this medium change, the medium was replaced again with serum-free culture medium containing samples. At first, NDI-1 was tested on U373 cells at 0.1, 1, 10, and 20 μM concentrations to identify the minimum cytotoxic concentration to be used in further experiments with SFNs (0.1 mg/mL); other SFNs concentrations were also tested (0.05 and 0.01 mg/mL). After an incubation of 24 and 48 h for the active, and 24 and 72 h for the nanoparticles, the supernatants were discarded, cells were washed with PBS, and 20 μL of MTS solution was added to 100 μL of fresh medium in each well. After 1.5 h of incubation, the plates were read at 490 nm by a Synergy HT plate reader (BioTek, Swindon, UK). The experiments were performed three times in quadruplicate, and the cell metabolic activity was calculated as in the following Equation (3):

Cell metabolic activity % = Abs_sample_/Abs_control_ × 100 (3)

where Abs_sample_ is the absorbance of the tested samples and Abs_control_ is the absorbance of cells not treated with any samples.

### 2.6. Statistical Analysis

The software STATGRAPHICS XVII (Statpoint Technologies, Inc., Warrenton, VA, USA) was used to elaborate raw data. As all data presented a normal distribution, a linear generalized analysis of variance model (ANOVA) was used, and the differences between the groups were evaluated with Fisher’s least significant difference (LSD) procedure. Statistical significance was set at *p* < 0.05.

## 3. Results and Discussion

Tetrasubstituted and tetracationic NDIs are well-known for being excellent G-quadruplex ligands, with a considerable selectivity towards these secondary structures concerning single- and double-stranded nucleic acid sequences [12]. However, their molecular structure and the high positive charge often make their selectivity for tumor cells negligible compared to normal cells. Therefore, identifying a new efficient delivery system to implement NDIs-specific anticancer activity is a groundbreaking proof-of-concept for these small molecules. In this context, we selected NDI-1, which showed a great specificity for G-quadruplex (ΔTm = 33.2 °C) over duplex DNA (ΔTm = 4.5 °C) with a good telomerase inhibition by TRAP assay with EC_50_ = 25 μM [42]. Nevertheless, although it produces short-term cell growth inhibition against the MCF7 and A549 cancer cell lines with IC_50_ values of 104.7 nM and 28.7 nM, respectively (by sulforhodamine B assay), the therapeutic window was very narrow, with a negligible selectivity over normal human fibroblast line WI38 (IC_50_ = 292 nM) [42]. Its synthesis was optimized, affording NDI-1 with a 37% overall yield against the 13% overall yield reported in the literature [42].

Silk fibroin nanoparticles were then prepared by inducing the coacervation of fibroin with acetone; therefore, starting from a homogeneous solution of fibroin and, in the case, NDI-1, at the end of the coacervation process, the active was entrapped in the fibroin matrix. The process yield was above 75% for all the batches prepared, and all the formulations were found to be nanometric in size, as reported in Table 2. In detail, following sonication and for all the formulations, the mean diameter was around or below 100 nm, and the d_90_ was always lower than 150 nm.

The fibroin matrix formed during the desolvation process could entrap the active compound, achieving a loading of 1.2 ± 0.4% *w*/*w*, with an encapsulation efficiency above 95%. Attempts were made to change the initial fibroin:NDI-1 ratio to gain SFNs with higher theoretical loading, but the actual drug loading was never above 1% *w*/*w*. It was supposed that NDI-1, a rigid, planar, and aromatic structure, can bind and settle only in the amorphous portions of fibroin chains that are more flexible, thus accommodating and firmly binding a flattened molecule such as NDI-1. It is likely that, after desolvation, fibroin is mainly present in the β-sheet form, which has a very compact texture, and thus there is not much space for NDI-1 in the matrix when the theoretical composition is above 1% *w*/*w*. Indeed, according to the literature [46,47], β-sheet regions were revealed in the IR spectra of SFNs by the absorption peaks in the amide I at around 1620 cm^−1^ (due to C=O stretching), precisely 1622.15 cm^−1^, the absorption peaks of amide II at around 1520 cm^−1^ (due to N-H bending), specifically at 1515.18 cm^−1^, and the peak of the amide II band at around 1266 cm^−1^ (due to C-N and N-H functionalities), specifically at 1260.89 cm^−1^ (Figure S2 in the Supplementary Materials).

**Scheme 2:**
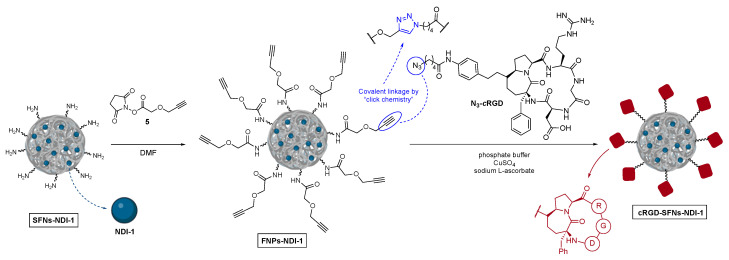
Preparation of alkyne-modified silk fibroin nanoparticles and their functionalization with azide-*c*RGDs through click chemistry.

The preparation of *c*RGD-functionalized SFNs (*c*RGD-SFNs and *c*RGD-SFNs-NDI-1) was performed by exploiting an azide-alkyne “click reaction” between the azide group of the RGD-based cyclic pentapeptide and triple bond-functionalized SFNs (Scheme 2). The functionalized nanoparticles (FNPs), carrying an alkyne group on their surface were obtained through forming an amide bond between amino groups on SFNs surface and N-hydroxysuccinimide linker 5 endowed with a terminal alkyne moiety. Lysine residues, 80 mmol/mg of protein on average [39], were chosen as the linker’s anchoring point. The obtained FNPs were then submitted to dialysis (cut-off 3–5 kDa MWCO) to remove reactants in excess and side products. The critical step, i.e., the Huisgen cycloaddition between FNPs and N3-*c*RGD, was accomplished in a phosphate buffer in the presence of CuSO_4_ and sodium l-ascorbate. The final dialysis (cut-off 3–5 kDa MWCO) of the reaction mixture afforded pure *c*RGD-SFNs or *c*RGD-SFNs-NDI-1.

The functionalization with *c*RGDs did not change the main characteristics of the nanoparticles. Following functionalization, the loading of the active compound was preserved (1.2 ± 0.3% *w*/*w*), as well as the nanometric size (Table 2). The data on the loading indirectly demonstrates that NDI-1 encapsulated in SFNs is not released from the protein matrix, even after sonication and 48 h of dialysis. As SFNs are prepared by coacervation, the active is mostly intrinsically entrapped in the protein matrix and only minimally exposed or adsorbed on the surface. Therefore, the release of most of NDI-1 from SFNs is not possible by desorption but only by diffusion through the fibroin matrix or following the biodegradation of the protein structure. The IR spectra showed that SFNs are rich in β-sheet form, which is very compact, and thus the active with a molecular weight of 960.49 g/mol can most likely diffuse through the nanoparticles’ matrix very slowly, making the diffusion process undoubtedly longer than the internalization by cells (which generally occurs in vitro in less than 30 min [40,48]). Similarly, the biodegradation of fibroin is much slower than the internalization time. In this way, NDI-1 should remain entrapped in SFNs while they circulate through the whole body, for example, following intravenous administration, and will be released only following the nanoparticle’s degradation in the cells’ cytosol.

Conversely, the zeta potential of SFNs changed after loading NDI-1 and functionalization with *c*RGDs, as reported in Table 3.

According to findings published in previous works [40], the functionalization of SFNs with *c*RGDs confers a negative surface charge to the nanoparticles; this effect was particularly evident after the functionalization of SFNs-ND-1, as the zeta potential changed from positive to markedly negative. The slightly positive charge of SFNs-NDI-1 is in agreement with our hypothesis about the minimum exposure of the active on the surface of the nanoparticles (vide supra) and explains why flocculation was observed during the dialysis process (see Figure S3 in Supplementary Materials). Indeed, the lower repulsive forces between nanoparticles allowed them to progressively approach until reaching the secondary minimum point in the DLVO theory [49]. At such a distance, the attractive forces are higher than the repulsive ones, so aggregates are formed. However, since SFNs are hydrophilic, they maintain a hydration film on their surface which prevents particles from collapsing. Therefore, SFNs formed non-compact and easily dispersible aggregates (flocs).

**Figure 1:**
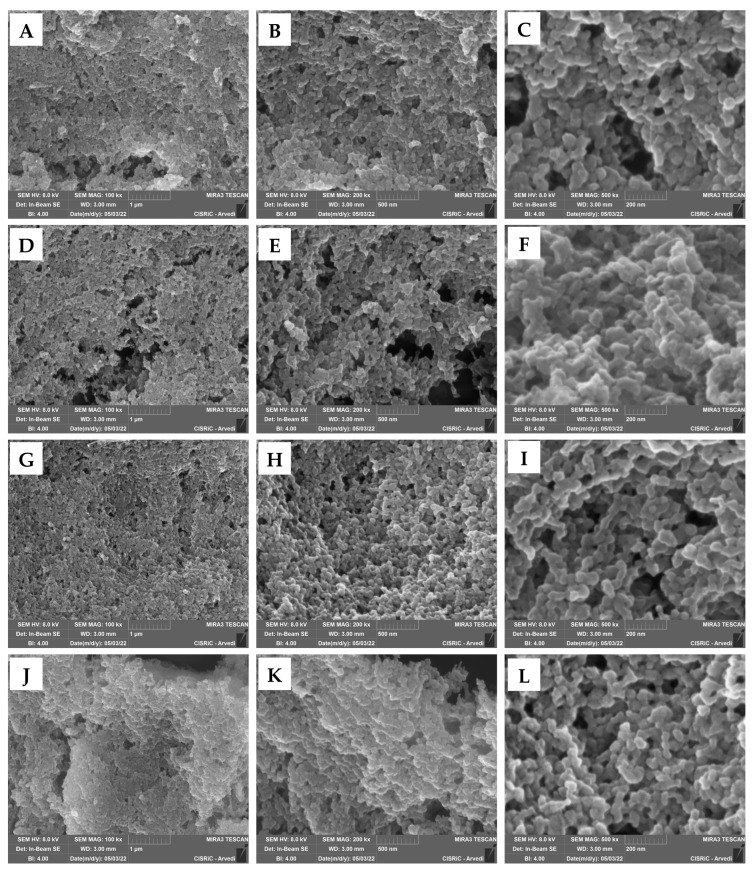
Morphological investigation by SEM of SFNs (**A**–**C**), SFNs-NDI-1 (**D**–**F**), *c*RGD-SFNs (**G**–**I**), and *c*RGD-SFNs-NDI-1 (**J**–**L**). Magnification: 100 k× (**A**,**D**,**G**,**J**), 200 k× (**B**,**E**,**H**,**K**), and 500 k× (**C**,**F**,**I**,**L**). Scale bar: 1 μm (**A**,**D**,**G**,**J**), 500 nm (**B**,**E**,**H**,**K**), and 200 nm (**C**,**F**,**I**,**L**).

**Figure 2:**
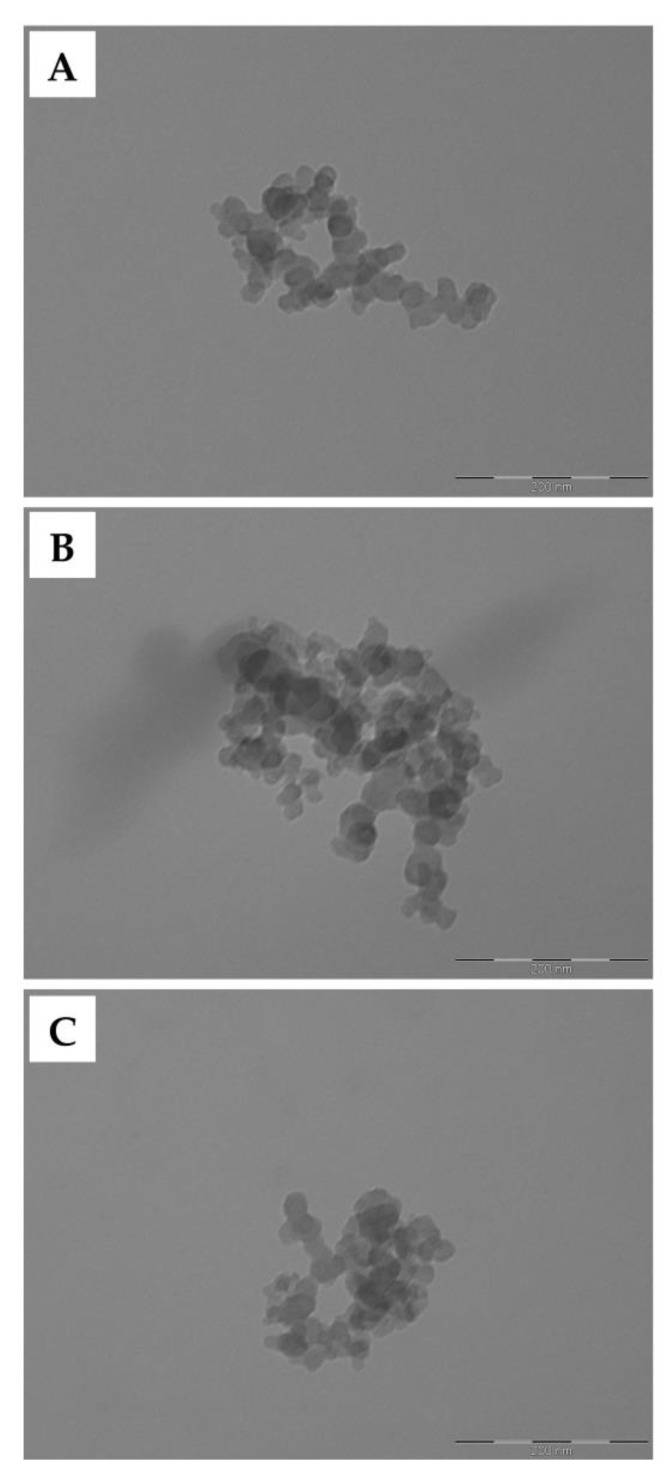
Morphological investigation by TEM of SFNs (**A**), SFNs-NDI-1 (**B**), and *c*RGD-SFNs-NDI-1 (**C**). Scale bar: 200 nm.

**Figure 3:**
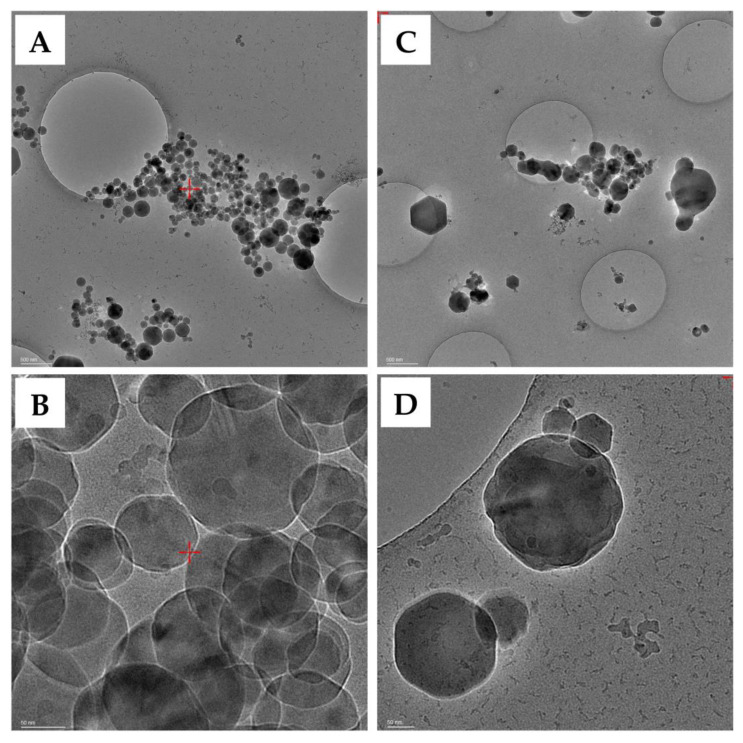
Ultrastructure investigation by cryo-EM of SFNs (**A**,**B**) and *c*RGD-SFNs-NDI-1 (**C**,**D**). Scale bar: 500 (**A**,**C**) and 50 (**B**,**D**) nm.

All samples observed by SEM were shown to be highly homogeneous in size and appearance (Figure 1). In detail, the nanoparticles displayed a round shape and a smooth surface. The exact morphology was then observed in more detail by TEM, confirming the same round morphology and smooth surface (Figure 2). Finally, cryo-EM images were acquired to observe the ultrastructure and the surface of the nanoparticles in more detail (Figure 3). All formulations appeared as a uniform matrix without holes or cavities. No appreciable differences were observed on the surface of the nanoparticles following functionalization. All of the morphological and ultrastructure analyses confirmed the nanometric size of all the samples, with a mean diameter around or lower than 100 nm in accordance with the data reported in Table 2.

Next, the cytotoxic effect of nanoparticles was investigated in vitro, specifically on the human glioma U373 and D384 cell lines. Firstly, the integrin expression pattern in the selected cell lines was evaluated by real-time qRT-PCR analysis. The data in Table 4 show that in D384 cells, αv, β3, and β5 mRNA was not detectable (ND, Cq > 41), while α5 and β1 mRNAs were expressed. In U373, the integrin expression pattern was quite the opposite, with the abundant expression of αv, β3, and β5 integrins. Since the *c*RGDs bind to αvβ3 integrins with high affinity, these data show that these two cell lines represent a very suitable model to study *c*RGDs-integrin interactions, D384 being a negative control for αvβ3 integrin expression and *c*RGDs binding. Data are expressed as ΔCq (difference between reference and housekeeping gene Cq).

**Figure 4:**
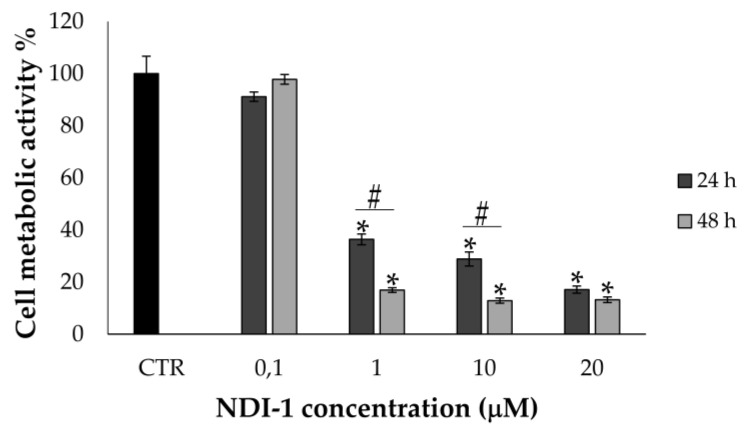
Cell metabolic activity % of U373 cells treated with increasing doses of NDI-1 for 24 and 48 h. Untreated cells at 24 h were considered CTR (100% of metabolic activity). Multifactor ANOVA, mean values ± least significant difference (LSD), n = 4. * *p* < 0.01 with respect to CTR-; # *p* < 0.05.

Subsequently, the active compound NDI-1 was tested on U373 cells at different concentrations (0.1, 1, 10, and 20 μM) and time of contact (24 and 48 h) to identify the minimum effective concentration and time. A concentration of 1 μM induced a marked decrease in cell viability after 24 h of contact (Figure 4).

Based on this information, to verify if the encapsulation of the active into nanoparticles reduced the toxicity in the short term and if the *c*RGD functionalization made the nanosystem selective, all SFNs formulations were tested. The tests were performed on the U373 cell line, overexpressing αv, β3, and β5 integrin subunits, and the D384 cell line, expressing these integrin subunits in amounts markedly lower than U373, as assessed by real-time RT-PCR analysis. At first, a dose-dependent effect for SFNs loaded with NDI-1 was observed: for both cell lines, the cytotoxic effect increased after increasing the tested concentration (see Figure S4A in the Supplementary Materials). As expected, only the concentration of 0.1 mg/mL, corresponding to 1 μM of NDI-1, significantly lowered the cell metabolic activity of both cell lines below 50%. Thereafter, it was observed that time also had a significant effect, and only exposure for 72 h was able to significantly lower the cell metabolic activity below 50%; indeed, after 24 h, all cells were quite viable, with a cell metabolic activity above 85% (see Figure S4B in the Supplementary Materials). This is in agreement with the above findings on the active release, confirming that the drug release can only be observed after internalization and degradation of the nanoparticles by the enzymes, thus requiring extended time before the cytotoxic effect is detectable.

**Figure 5:**
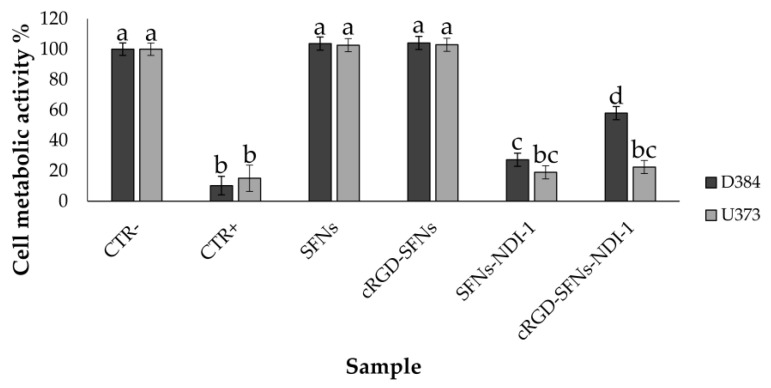
Cytotoxic effect of samples tested at 0.1 mg/mL on U373 (expressing integrin subunits) and D384 (expressing integrin subunits in lower amounts) cell lines after 72 h. Untreated cells were considered CTR- (100% of metabolic activity), while 1 μM of NDI-1 was considered CTR+. Multifactor ANOVA, mean values ± least significant difference (LSD), n = 4 from two independent experiments. Letters (a, b, bc, c, and d) are used to compare the means of different groups. If the letters are different, there is a statistically significant difference between the means (*p* < 0.05); if the letters are the same, there is no statistically significant difference between the means (*p* > 0.05).

After 72 h of treatment, unloaded SFNs, either functionalized with *c*RGD or not, showed cytocompatibility, with a cell metabolic activity close to 100% for both cell lines (Figure 5). Conversely, NDI-1-loaded SFNs were highly cytotoxic on both D384 and U373 cells, reducing the metabolic activity below 30% and 20%, respectively. The toxic effect of SFNs-NDI-1 showed to be slightly lower with respect to the free active, and it was not selective for a specific cell line. Therefore, these data suggest that both cell lines can internalize SFNs-NDI-1 by non-specific mechanisms (likely favored by the small positive charge of these nanoparticles); NDI-1 exerts its cytotoxic effect after the internalization and degradation of SFNs. Functionalization with *c*RGD provided selectivity in cell uptake, probably coupling non-specific cell membrane protein–particle interactions to specific and target-mediated uptake. As a result, *c*RGD-SFNs-NDI-1 was highly cytotoxic on U373 cells, while a significantly weaker effect was observed on D384 cells (a metabolic activity of 22% versus 58%). The effectiveness of the functionalization in providing selectivity is further supported by the zeta potential data (Table 3): despite the repulsive effect between *c*RGD-SFNs-NDI-1 and the cell membrane (both negatively charged), the internalization by the U373 cell and the resulting toxic effects are marked. This is probably due to the overexpression of integrin receptors, which counteracted and overcame the unfavorable effects of electrostatic repulsion.

Overall, the formulated nanoparticles had the following characteristics: (i) morphology, size, and surface charge that make them optimal for cell uptake; (ii) ability to load up to 1% *w*/*w* of NDI-1 with a high encapsulation efficiency (over 95%); (iii) the loaded active is retained by the protein matrix of fibroin, and it is released and exerts its cytotoxic effect only after internalization and degradation of SFNs in the cytosol; (iv) the functionalization with *c*RGDs made the uptake of SFNs by cells that overexpress αvβ3 and αvβ5 integrin receptors selective.

## 4. Conclusions

In this work, compound NDI-1, a tetrasubstituted naphthalene diimide derivative with potent cytotoxic anticancer activity, was encapsulated into SFNs with a high encapsulation efficiency. Active targeting was provided for SFNs by functionalizing their surface with cyclic pentapeptides bearing the RGD sequence (*c*RGDs), making the uptake by cells that overexpress αvβ3 and αvβ5 integrin receptors selective. The encapsulation of NDI-1 into *c*RGD-SFNs could prevent it from exerting systemic cytotoxic effects. Moreover, following accumulation in the tumor (guided by the EPR passive targeting and active uptake by tumor cells), the high cytotoxicity of NDI-1 could be selectively directed towards malignant cells overexpressing αvβ3 and αvβ5 integrin receptors. Therefore, this manuscript provides the in vitro proof-of-concept of cRGD-SFNs active site-specific targeting for tumor site-specific delivery, paving the way for in vivo studies and in vitro innovative models as alternatives to animal use.

## Figures and Tables

**Table 1:** Primers used to amplify RGD-binding integrin mRNA in real-time qRT-PCR experiments.

Gene	Accession Number	Primer Sequence
αv	NM_002210	F: actggcttaagagagggctgtg R: tgccttacaaaaatcgctga
β3	NM_000212	R: tcctcaggaaaggtccaatg R: tcctcaggaaaggtccaatg
β5	NM_002213	F: agcctatctccacgcacact R: cctcggagaaggaaacatca
α5	NM_002205	F: cctgctgtccaccatgtcta R: ttaatggggtgattggtggt
β1	NM_133376	F: tccaatggcttaatttgtgg R: cgttgctggcttcacaagta
β-actin	NM_001101.5	R: atgatctgggtcatcttctc F: gacgacatggagaaaatctg

**Table 2:** Size and size distribution of all the nanoparticles prepared. Data are reported in nm as mean value ± standard deviation, n = 5 independent measurements.

Formulation	Mean Diameter	Mode	d_10_	d_50_	d_90_
SFNs	73.2 ± 3.5	87.8 ± 1.3	41.9 ± 10.3	82.9 ± 0.3	92.5 ± 0.9
*c*RGD-SFNs	64.6 ± 6.0	40.3 ± 3.9	35.5 ± 4.2	53.2 ± 3.9	106.7 ± 10.9
SFNs-NDI-1	100.6 ± 3.6	102.4 ± 0.9	83.4 ± 8.3	101.3 ± 1.2	110.7 ± 4.6
*c*RGD-SFNs-NDI-1	78.9 ± 2.2	60.8 ± 0.6	55.4 ± 0.4	64.2 ± 0.8	138.8 ± 20.9

**Table 3:** Zeta potential of all the nanoparticles prepared. Data are reported in mV as mean value ± standard deviation, n = 3 independent measurements.

Formulation	Zeta Potential
SFNs	−20.3 ± 2.40
*c*RGD-SFNs	−10.4 ± 0.86
SFNs-NDI-1	+3.97 ± 0.40
*c*RGD-SFNs-NDI-1	−29.4 ± 0.21

**Table 4:** Expression of integrin subunits in U373 compared to D384 cells. Data are expressed as ΔCq.

	αv	α5	β1	β3	β5
U373	6.11	6.33	3.14	7.86	8.25
D384	21.99	8.81	10.31	ND (Cq > 41)	ND (Cq > 41)

## Data Availability

The data presented in this study are contained within the article.

## Supplementary Materials

The following supporting information can be downloaded at: https://www.mdpi.com/article/10.3390/cancers15061725/s1, Figure S1: Analytic HPLC profile of pure NDI-1 (rT = 4.996 min, 96.2%); Figure S2: Representative FTIR spectra for SFNs; Figure S3: Flocculation of SFNs-NDI-1 during dialysis (A) progressively leads to flocked sediment formation (B). Figure S4: Effect of concentration (A) and time (B) on cell metabolic activity. Data are reported as mean values of the cell metabolic activity of both cell lines ± LSD, n = 4.
