# Supplementary material for: cRGD-Functionalized Silk Fibroin Nanoparticles: A Strategy for Cancer Treatment with a Potent Unselective Naphthalene Diimide Derivative
Source: Cancers (Basel). 2023 Mar 11;15(6):1725. doi: 10.3390/cancers15061725 (PMC10046852; doi:10.3390/cancers15061725)
Supplement: Supplementary file 1 [file cancers-15-01725-s001.zip › cancers-2151220-supplementary.pdf]

Supplementary materials

Silk Fibroin Nanoparticles Functionalized with RGD-based Cyclic Pentapeptide: a "Magic Bullet" for a Potent Unselective Anticancer Naphthalene Diimide Derivative

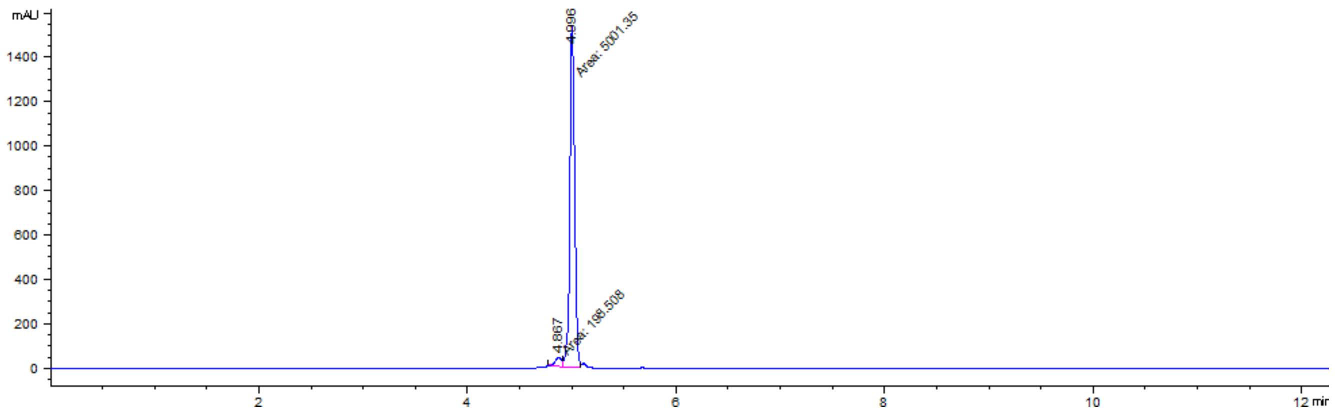

Figure S1. Analytic HPLC profile of pure NDI-1 (rT = 4.996 min, 96.2%).

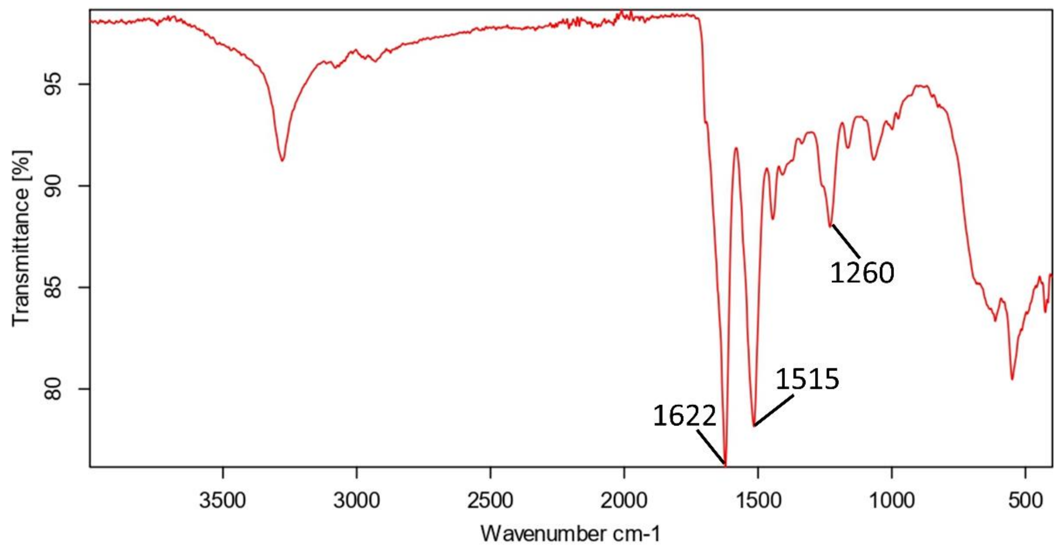

Figure S2. Representative FTIR spectra for SFNs

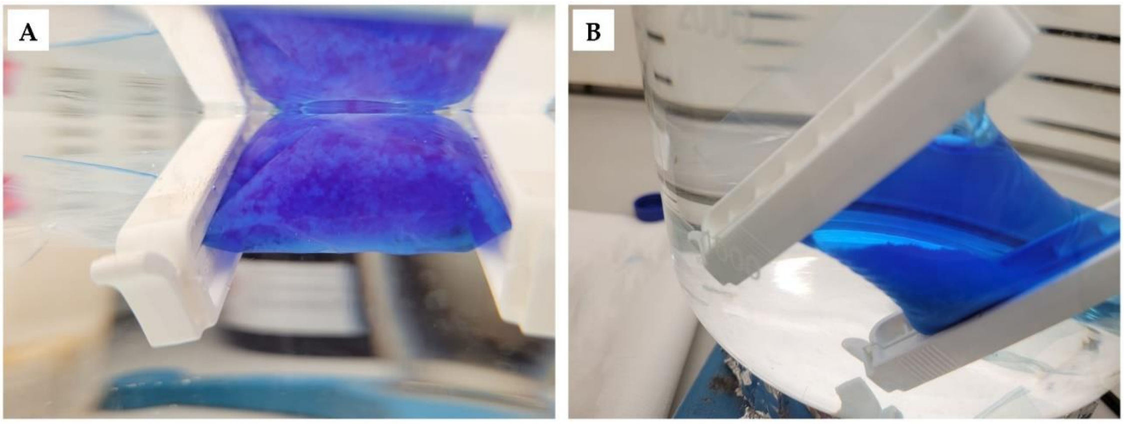

Figure S3. Flocculation of SFNs-NDI-1 during dialysis (A) progressively leads to flocked sediment formation (B).

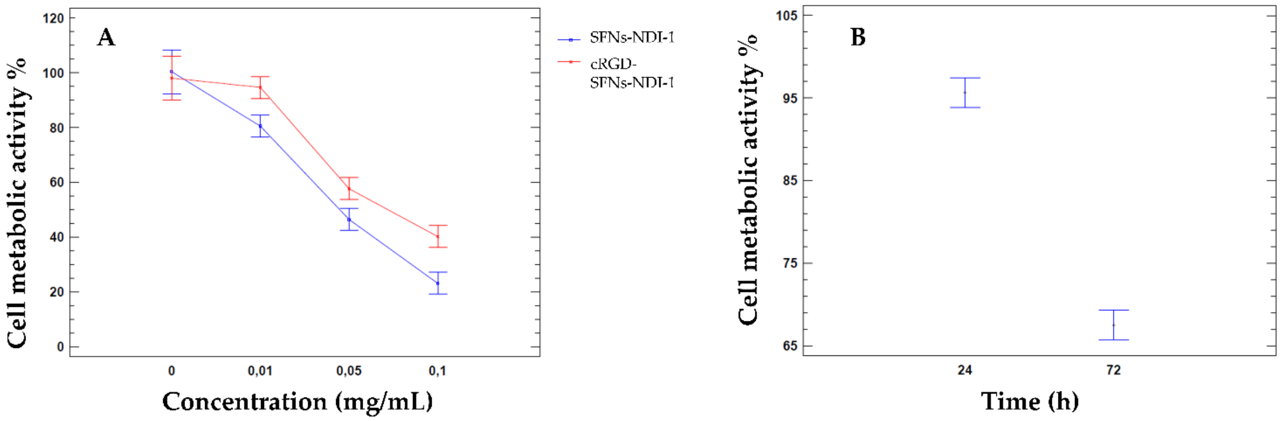

Figure S4. Effect of concentration (A) and time (B) on cell metabolic activity. Data are reported as mean values of the cell metabolic activity of both cell lines ± LSD, n=4
